# Identifying the genes of unconventional high temperature superconductors

**DOI:** 10.1007/s11434-016-1037-7

**Published:** 2016-03-14

**Authors:** Jiangping Hu

**Affiliations:** Beijing National Laboratory for Condensed Matter Physics, Institute of Physics, Chinese Academy of Sciences, Beijing, 100190 China; Collaborative Innovation Center of Quantum Matter, Beijing, 100190 China

**Keywords:** Cuprates, Iron-based superconductors, Unconventional high *T*_c_ superconducotors, Superexchange

## Abstract

We elucidate a recently emergent framework in unifying the two families of high temperature (high $T_{\rm c}$) superconductors, cuprates and iron-based superconductors. The unification suggests that the latter is simply the counterpart of the former to realize robust extended s-wave pairing symmetries in a square lattice. The unification identifies that the key ingredients (gene) of high $T_{\rm c}$ superconductors is a quasi two dimensional electronic environment in which the *d*-orbitals of cations that participate in strong in-plane couplings to the *p*-orbitals of anions are isolated near Fermi energy. With this gene, the superexchange magnetic interactions mediated by anions could maximize their contributions to superconductivity. Creating the gene requires special arrangements between local electronic structures and crystal lattice structures. The speciality explains why high $T_{\rm c}$ superconductors are so rare. An explicit prediction is made to realize high $T_{\rm c}$ superconductivity in Co/Ni-based materials with a quasi two dimensional hexagonal lattice structure formed by trigonal bipyramidal complexes.

## 摘要

八年前铁基超导体的偶然发现带来了高温超导新–轮研究高潮的同时,也不得不让 我们深刻地反思过去近三十年铜基高温超导的研究的问题:为什么过去的研究没有 对铁基超导体发现提供任何有意义的线索?究竟是什么特性使得它们独立于其他材 料成为高温超导体?本文基于对两者的超导配对对称性统–理解出发提出两者的配 对机理都来自反铁磁超交换相互作用并且指出两者都拥有支持该机理的独特电子结 构–在费米能量附近,参与强反铁磁超交换相互作用的电子轨道独立存在.这种独 特电子结构可以被认为是高温超导的基因.它们的形成是由构造材料的基本配位单 元,材料整体晶格结构以及轨道上的电子数三者共同配合决定的.本文预言这种基 因还可以在由三角双锥配位单元组合的二维六方晶格中当d 轨道上有7 个电子(Co^2+^/Ni^3+^) 附近实现,并且会支持d+id 的超导配对。因此合成这种结构的材料会可能 对非常规高温超导机理有决定性的影响。

## Introduction

Almost three decades ago, the first family of unconventional high $T_{\rm c}$ superconductors, cuprates [1], was discovered. The discovery triggered intensive research and has fundamentally altered the course of modern condensed matter physics in many different ways. However, even today, after tens of thousands of papers devoted to the materials have been published, understanding their superconducting mechanism remains a major open challenge. Researchers in this field are sharply divided and disagree with each other on many issues arranging from minimum starting models to basic physical properties that are relevant to the cause of superconductivity. There is even a growing skepticism whether there are right questions that can be asked to settle the debate on the superconducting mechanism.

Many reasons can be attributed to the failure of answering the question of how superconductivity arises in cuprates. For example, material complexity makes theoretical modeling difficult, rich physical phenomena blind us from distinguishing main causes from side ones, and insufficient theoretical methods leave theoretical calculation doubtable. However, beyond all these difficulties and the absence of consensus, the lack of successfully realistic guiding principles to search for new high $T_{\rm c}$ superconductors from theoretical studies is the major reason. The failure was witnessed in the surprising discovery of the second family of high $T_{\rm c}$ superconductors, iron-based superconductors [2], in 2008. Today, those who are theory builders and those who are material synthesizers still remain disentangled.

Can valuable leads be provided from the theoretical side ahead of the potential discovery of the third family of high $T_{\rm c}$ superconductors? It is conceivable that the hope to settle high $T_{\rm c}$ mechanism relies on a positive answer to this question. Here, we believe that it is the time to seek a positive answer based on the following two reasons. First, in the past 7 years, the intensive research on iron-based superconductors has brought much new information. For those who believe that cuprates and iron-based superconductors should share a common high $T_{\rm c}$ mechanism, an opportunity to settle the debate arises as it is the first time that the traditional inductive reasoning becomes available in research. On one side, iron-based superconductors and cuprates share many common features, but on the other side they are not clones of each other. The similarities and differences can thus speak promising clues. Second, from the past massive searching efforts, it has become increasingly clear that unconventional high $T_{\rm c}$ superconductors are rare materials. Moreover, for the two known families, their superconductivities are carried robustly on $\hbox {CuO}_2$ layers in cupates and on FeAs/Se layers in iron-based superconductors respectively. The simultaneous existence of the rareness and robustness suggests that the unconventional high $T_{\rm c}$ superconductivity is tied to special ingredients in the electronic world, which define the gene of unconventional high $T_{\rm c}$ superconductivity. Thus, using inductive reasoning to identify the gene can open a new window to search for high $T_{\rm c}$ superconductors.

In this article, by taking the assumption that a common superconducting mechanism is shared by both known high $T_{\rm c}$ superconductors, we elucidate a recently emergent path to end the deadlock in solving high $T_{\rm c}$ mechanism by implementing inductive reasoning to reexamine the high $T_{\rm c}$ problem [3, 4]. This path stems from a simple framework that unifies cuprates and iron-based superconductors based on previous understandings in repulsive interaction or magnetically driven high $T_{\rm c}$ mechanisms. It suggests that iron-based superconductors are simply the counterpart of cuprates to realize robust extended s-wave pairing symmetries in a square lattice. Both materials share a key ingredient, the gene of unconventional high $T_{\rm c}$ superconductivity: a quasi two dimensional electronic environment in which the *d*-orbitals of cation atoms that participate in strong in-plane couplings to the *p*-orbitals of anion atoms are isolated near Fermi energy. This environment allows the antiferromagnetic (AFM) superexchange couplings mediated through anions, the source of superconducting pairing, to maximize their contributions to superconductivity. Creating such a gene is tied to special arrangements between local electronic structures and crystal lattice structures, which explains why cuprates and iron-based superconductors are special and high $T_{\rm c}$ superconductors are so rare. The framework can be explicitly tested in future experiments as it leads to an explicit prediction to realize high $T_{\rm c}$ superconductivity in the Co/Ni-based materials with a quasi two dimensional hexagonal lattice structure formed by trigonal bipyramidal (TBP) complexes [3]. The new materials are predicted to be high $T_{\rm c}$ superconductors with a $d\pm {\mathrm{i}}d$ pairing symmetry. If verified, the prediction will establish powerful guiding principles to search for high $T_{\rm c}$ superconductor candidates, as well as to settle the debate on unconventional high $T_{\rm c}$ superconducting mechanism.

## Questions for unconventional high $T_{\rm c}$ superconductivity

Implementing inductive reasoning to understand both cuprates and iron-based superconductors, we lay out the high $T_{\rm c}$ problem with the following three subsequent questions:

1. What is the common interaction responsible for high $T_{\rm c}$ superconductivity in both families?
2. What are the key ingredients to make both families special to host high $T_{\rm c}$ superconductivity?
3. Where and how can we search for new high $T_{\rm c}$ superconductors?

The three questions are highly correlated. They form a self-contained unit to reveal high $T_{\rm c}$ superconducting mechanism.

In the past, the first question was the central question. Its answer was debated wildly. The second question was largely ignored. However, after the discovery of iron-based superconductors, it becomes clearer that the second question should be the central piece. While most researches have concentrated on these two families of high $T_{\rm c}$ superconductors, it is equally important to answer why numerous materials, which are similar to cuprates or iron-based superconductors in many different ways, do not exhibit high $T_{\rm c}$ superconductivity. Therefore, the essential logic here is that whatever our answer to the first question is, the answer must provide an answer to the second question. The answer to the second question can provide promising leads to answer the third question. An explicit theoretical prediction of new high $T_{\rm c}$ superconductors and its experimental verification can finally justify the answer of the first question to end the debate on high $T_{\rm c}$ mechanism.

## The ansatz to the first question

We start with the first question. Our proposed answer to the first question is that only the superexchange AFM interactions mediated through anions are responsible for generating superconductivity in both families of high $T_{\rm c}$ superconductors. We call this ansatz as the selective magnetic pairing rule [4] in the repulsive interaction or magnetically driven superconducting mechanisms. One may argue that this answer is somewhat trivial as it has been accepted in a variety of models for cuprates [5, 6]. However, as we will discuss below, the answer is highly non-trivial in iron-based superconductors because their magnetisms are involved with different microscopic origins. Three main reasons to support this rule are:

1. It naturally explains the robust d-wave pairing symmetry in cuprates and the robust s-wave pairing symmetry in iron-based superconductors;
2. It is supported by a general argument that without the existence of mediated anions in the middle, the short-range Coulomb repulsive interactions between two cation atoms can not be sufficiently screened to allow superconducting pairing between them;
3. It places strict regulations on electronic environments that can host high $T_{\rm c}$ superconductivity and thus results in a straightforward answer to the second question.

### The case of cuprates

As we have pointed out above, the rule is a familiar ansatz in cuprates. It has provided a natural explanation to the d-wave pairing symmetry [7], arguably the most successful theoretical achievement in the studies of cuprates. In fact, historically, in determining the pairing symmetry of cuprates, the d-wave pairing symmetry was theoretically predicted before the emergence of major experimental evidence [8–10].

**Fig. 1:**
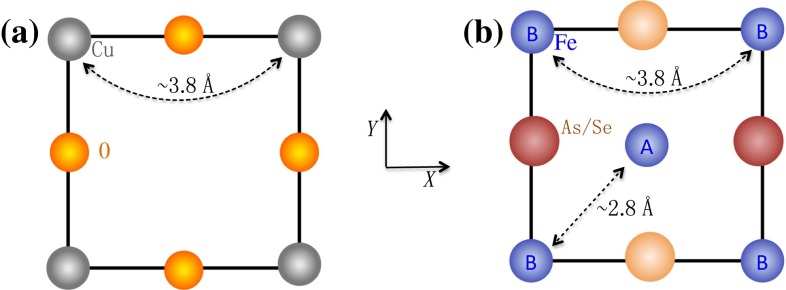
(Color online) The comparison of the lattice unit cells between cuprates and iron-based superconductors. **a** The unit cell and lattice constant of the $\hbox {CuO}_2$ layer in cuprates; **b** The unit cell and lattice constant of the FeAs/Se layer which includes two irons marked as A and B

Here we briefly review the main theoretical approaches in obtaining the d-wave pairing symmetry in curpates. There are two types of approaches to obtain the d-wave pairing symmetry based on effective models built in a two-dimensional Cu square lattice (Fig. 1a). One is the traditional weak coupling approach. This approach starts with a closely nested Fermi surfaces in which the spin-density wave (SDW) instability can take place by onsite electron–electron repulsive interaction (the Hubbard interaction) [7, 8]. The other is the strong interaction approach. It starts directly with short-range magnetic exchange interactions. In cuprates, the magnetic exchange interactions are the nearest neighbor (NN) AFM superexchange interactions mediated through oxygen atoms [5, 9, 10]. Both approaches consistently predict d-wave superconducting states.

**Fig. 2:**
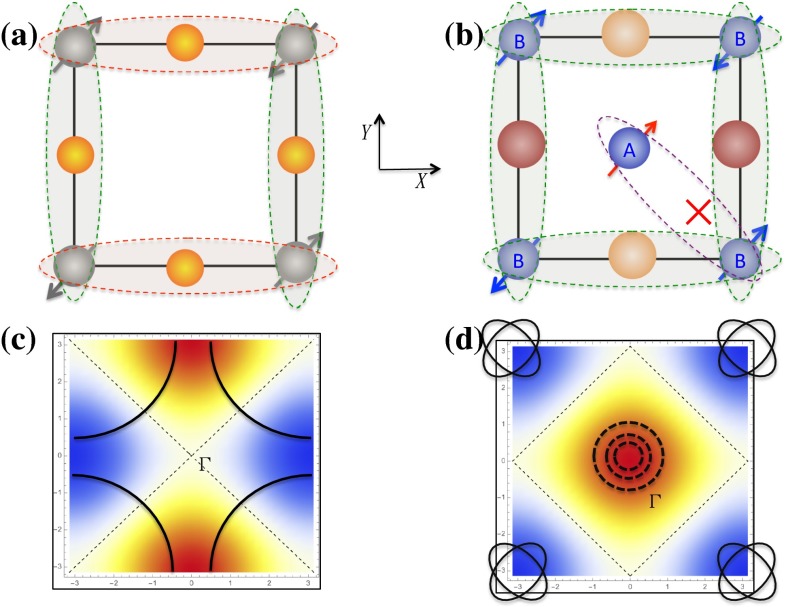
The comparison of superconducting pairings between curpates and iron-based superconductors in both real and momentum spaces. **a** The real space pairing configuration in the d-wave superconducting state of cuprates; **b** The real space pairing configuration in the extended s-wave superconducting state in iron-based superconductors (the red multiplication sign indicates the forbidden pairing between A and B sublattices); **c** The Fermi surfaces of cuprates and the weight distribution of the d-wave order parameter in the momentum space (red and blue colors represent regions with large positive and negative values respectively); **d** The typical Fermi surfaces of iron-based superconductors and the weight distribution of the extended s-wave order parameter in the momentum space. The Fermi surfaces at $\Gamma$ with dashed lines are hole pockets which can be absent in iron-chalcogendies [13]

The consistency can be attributed to the following simple pairing symmetry selection rule: the pairing symmetry is selected by the weight of its momentum space form factor on Fermi surfaces [11]. This rule is based on the following observation in repulsive interaction or magnetically driven high $T_{\rm c}$ superconducting mechanism: the superconducting pairings are dominated on bonds with the strongest effective AFM exchange couplings. This rule has been emphasized in the second type of models with local AFM superexchange interactions [10, 12]. In the case of cuprates, the decoupling of the NN AFM superexchange interaction in the pairing channel results in two possible pairing symmetries: an extended s-wave with a superconducting order in the reciprocal space $\Delta _{\mathrm{s}}(k)\propto {\mathrm{cos}}k_X+{\mathrm{cos}}k_Y$ and a d-wave with $\Delta _{\mathrm{d}} (k)\propto {\mathrm{cos}}k_X-{\mathrm{cos}}k_Y$. With the Fermi surface shown in Fig. 2c, the d-wave form factor has a much larger amplitude on the Fermi surfaces than the extended s-wave. Thus, the d-wave is favored by opening much larger superconducting gaps to save more AFM exchange energy in the superconducting state. This rule is also behind the weak coupling approach based on the Hubbard model in cuprates [7]. As the Hubbard model only includes the onsite repulsive interactions and its kinetic part is dominated by the NN hopping, the leading effective AFM exchange couplings are also generated on the NN bonds. In fact, considering the AFM fluctuations near half-filling in the Hubbard model, the effective electron–electron interaction mediated by the AFM fluctuations in the pairing channel has the following property [7]: it starts with a large repulsive onsite interaction followed by an attractive interaction between two NN sites, and then oscillates between repulsive and attractive with a rapid decay as increasing the space distance. This property essentially tells us that the pairing is also dominated on the NN bonds.

### The case of iron-based superconductors

Comparing an FeAs/Se layer with a $\hbox {CuO}_2$ layer, as shown in Fig. 1a, b, we notice several important differences: (1) the As/Se atoms in the former are located exactly below or above the middle points of the four-Fe squares; (2) the distance between two NN Fe atoms is very short, which is only about 2.8 Å. This value is close to the lattice constant of the body-centered cubic Fe metal; (3) the distance between two next NN (NNN) Fe atoms is about 3.8 Å, which is close to the distance of two NN Cu atoms in the $\hbox {CuO}_2$ layer. These differences suggest that the magnetic exchange couplings between two NNN Fe atoms, just like those between two NN Cu atoms, are mediated by the *p*-orbitals of As/Se atoms. Thus the magnetic couplings between two NNN sites are dominated by superexchange mechanism. However, two *d*-orbitals between two NN Fe atoms have large overlap which causes direct hoppings and direct magnetic exchange couplings. Therefore, the NN exchange magnetic couplings have a different microscopic mechanism from the NNN ones. These differences explain why the effective magnetic models in iron-based superconductors are much complex and exhibit both itinerant and local types of magnetic characters [14].

The short NN distance and the existence of direct magnetic exchange mechanism also have a profound effect on the superconducting pairings. In cuprates, one can argue that the repulsive interaction between two NN Cu atoms can be ignored because of the existence of oxygen atoms in the middle, which create a large local electric polarization to screen the effective Coulomb interaction. This allows pairing to take place on the NN bonds. However, if there is a direct hopping between two atoms, there is no local electronic polarization to screen the Coulomb interactions between them. Thus, in iron-based superconductors, the repulsive interaction between two NN Fe sites must be large so that the pairing between NN bonds is essentially forbidden. But the physics between two NNN Fe sites are the same as those between two NN sites of cuprates. The effective Coulomb interaction between two NNN sites is screened by the strong electronic polarization created by As/Se atoms.

We can picture the above discussion in a simple manner. Considering the original two-iron unit cell as shown Fig. 1b, we label the two Fe sites in the unit cell as A and B respectively so that the Fe square lattice composes of two square sublattices, A and B. Each sublattice can be considered as an analogy of the Cu square lattice of cuprates, The pairings between the two lattices are forbidden due to the existence of strong repulsive interactions. The pairing exists only within each sublattice. Namely, as illustrated in Fig. 2b, the pairings are only allowed between different 2-Fe unit cells and are forbidden within the unit cells. Such an analogy allows us to apply the same pairing symmetry selection rules to predict the pairing symmetry of iron-based superconductors. If we draw the Fermi surfaces, as shown in Fig. 2d, in the Brillouin zone of the two Fe unit cell, which is also the Brillouin zone with respect to each sublattice, the Fermi surfaces are located either at the corner (*M*) or at the center $(\Gamma )$. As shown in Fig. 2d, the form factor of the extended s-wave $\Delta _{\mathrm{s}}(k)$ has a large weight on Fermi surfaces. Thus, the extended s-wave is clearly favored. The picture does not depend on the presence or absence of hole pockets at $\Gamma$ points.

The above discussion suggests that iron-based superconductors are simply a counterpart of cuprates to realize the extended s-wave pairing symmetry in a square lattice. The extended s-wave in iron-based superconductors endures the same robustness as the d-wave in cuprates. The robust s-wave symmetry in iron-based superconductors has been supported by overwhelming experimental evidence accumulated in the past several years [16, 16, 17]. This understanding explains the missing part in the previous theoretical studies which failed to obtain the robust s-wave pairing. In the previous studies based on weak coupling approaches [17], the repulsive interaction between the A and B sublattices is not seriously considered and only onsite repulsive interactions are considered in calculating pairing symmetries. With only onsite repulsive interaction, the effective attractive interactions are generated in both NN and NNN bonds. In general, the NN bonds favor the d-wave pairing symmetry [18] and the NNN bonds favor the extended s-wave symmetry. Thus, pairing symmetries from these models become very sensitive to the detailed parameters and Fermi surface properties [17, 18]. The same sensitivity also exists in the models based on local AFM $J_1{-}J_2$ exchange couplings [12]. With the existence of both $J_1$, the NN AFM exchange couplings, and $J_2$, the NNN AFM exchange couplings, the phase diagram is very rich [12, 19]. The robust s-wave is only obtained when $J_1$ is argued to be inactive in providing pairing [20].

Summarizing above discussions, iron-based superconductors and cuprates can be unified in one superconducting mechanism. The former provides extreme valuable information to distinguish the roles of different magnetic interactions in providing superconducting pairing. The robust s-wave pairing symmetry in iron-based superconductors, just like the d-wave in cuprates, is a strong indiction to support the AFM superexchange couplings as the dominant sources for pairing.

## The answer to the second question

As we have mentioned earlier, the challenge is that the answer to the first question has to result in a natural answer to the second question. To show that this is the case for the above ansatz, we first discuss explicit conditions posed by the answer to the first question. Then, we discuss how both cuprates and iron-based superconductors fulfill these conditions. Finally, we address why it is difficult to satisfy these conditions and explain why unconventional high $T_{\rm c}$ superconductors are rare.

### Conditions and rules for unconventional high $T_{\rm c}$ superconductivity

In order to generate the strong AFM superexchange couplings and maximize their contributions to high $T_{\rm c}$ superconductivity, we can argue the following specific requirements for potential high $T_{\rm c}$ candidates:

1. The necessity of cation–anion complexes: as the AFM superexchange couplings are mediated through anions, the potential candidates must include structural units constructed by cation–anion complexes. Within the units, there must be shared anions between two neighboring complexes. Moreover, strong chemical bondings between two anions should be forbidden as they generally destroy the AFM exchange processes.
2. The orbital selection rule: the orbitals of cation atoms that participate in strong chemical bondings with anion atoms to generate strong AFM superexchange couplings must play a dominant role near Fermi energy. The best electronic environment for high $T_{\rm c}$ superconductivity is achieved when these orbitals are isolated near Fermi energy. Namely, the band structures near Fermi energy should be dominated by the orbitals of cation atoms whose kinematics are generated through the couplings to anions. We will show that this requirement essentially answers why cuprates and iron-based superconductors are special to host high $T_{\rm c}$ superconductivity. It is the most powerful rule to narrow our search for potential high $T_{\rm c}$ candidates. Following this rule, we can combine symmetry analysis and density functional theory (DFT) to search for new high $T_{\rm c}$ electronic environments. This rule has been implicated in cuprates as an orbital distillation effect based on the observation that the higher $T_{\rm c}$ is achieved when $d_{X^2-Y^2}$-orbitals are dressed less by $d_{Z^2}$ orbitals in cuprates [21].
3. The pairing symmetry selection rule: we have explicitly discussed this rule above. This rule allows us to link pairing configurations in real and momentum spaces directly. Following this rule, we may be able to design structures to realize superconducting states with specific pairing symmetries.
4. Electron–electron correlation and half-filling: the atomic orbitals in cation atoms that can produce strong AFM superexchange couplings require to balance their spatial localization and extension. Moreover, in general, the strong AFM superexchange couplings are achieved when the orbitals are close to be half-filling. Thus, the half-filled 3*d* orbitals in transition metal elements are clearly the best choices.
5. Dimensionality: for *d*-orbitals, due to their two-dimensional nature in the spatial configuration, the orbital selection rule naturally demands a quasi two dimensional electronic environment. In an electronic band structure with strong three-dimensional band dispersions, it is difficult to maintain a purified orbital character. While one may argue that it is possible to satisfy these requirements in quasi one dimensional electronic environments, finding such an example is extremely difficult.

Summarizing these conditions and rules for transition metal based compounds, we can specifically define the gene of high $T_{\rm c}$ superconductors as a quasi two dimensional electronic structure in which the *d*-orbitals of cation atoms that participate strong in-plane chemical bonding with the *p*-orbitals of anion atoms are isolated near Fermi energy. In the following two subsections, we show that both cuprates and iron-based superconductors are special materials to carry such a gene.

### The case of curpates

**Fig. 3:**
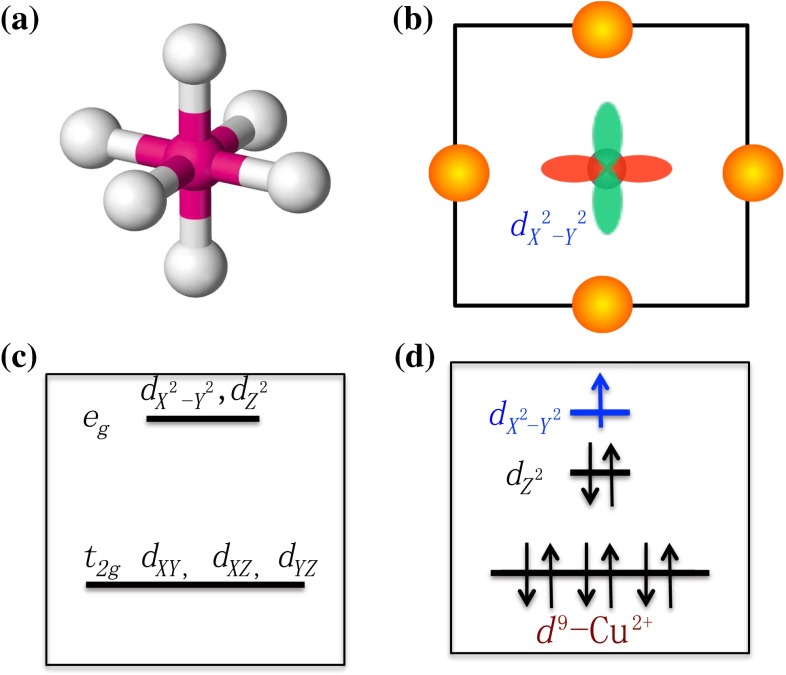
Local electronic environment and selected orbitals in cuprates. **a** The sketch of an octahedral complex; **b** The coupling configuration of the selected $d_{X^2-Y^2}$ orbital in $\hbox {CuO}_2$ layers; **c** The crystal field splitting of cation *d*-orbitals in an octahedral complex; **d** The true local energy configurations at Cu sites in curpates to indicate that the blue orbital, $d_{X^2-Y^2}$, is selected in the $d^9$ filling configuration

Cuprates belong to perovskite-related structural materials. The perovskite-related structures are the most popular and stable structures in nature. In a perovskite-related structure, the basic building block is the cation–anion octahedral complex shown in Fig. 3a. In cuprates, the $\hbox {CuO}_6$ octahedral complexes form two dimensional $\hbox {CuO}_2$ layers to provide a quasi two dimensional electronic structure. In a pure $\hbox {CuO}_6$ octahedral complex, the five *d*-orbitals of the Cu atom are split into two groups by crystal fields, $t_{2g}$ and $e_g$, as shown in Fig. 3c. The energies of the two $e_g$ orbitals, $d_{Z^2}$ and $d_{X^2-Y^2}$, due to their strong couplings to the surrounding oxygen atoms, are higher. Moreover, in the $\hbox {CuO}_2$ layer, the energy of $d_{Z^2}$ orbital is lowered either by the Jahn–Teller effect or by the absence of apical oxygen atoms. Thus, the local energy configuration at cation sites is described according to Fig. 3d in which the $d_{X^2-Y^2}$ orbital sits alone at the top.

It is easy to notice that only the $d_{X^2-Y^2}$ orbital has strong in-plane couplings to the *p*-orbitals of oxygens to mediate strong AFM superexchange couplings. Namely, only the electronic band attributed to the $d_{X^2-Y^2}$ orbital can support high $T_{\rm c}$ superconductivity. To isolate the $d_{X^2-Y^2}$ orbital near Fermi energy, nine electrons on the d shell are required. Thus, the gene of high $T_{\rm c}$ superconductivity can only be satisfied in a $d^9$ filling configuration at cation sites, which explains why Cu$^{2+}$ is a natural choice. As a matter of fact, in the past several decades, numerous transition metal compounds with perovskite-related structures were discovered. Except curpates, none of them exhibits high $T_{\rm c}$ superconductivity.

### The case of iron-based superconductors

**Fig. 4:**
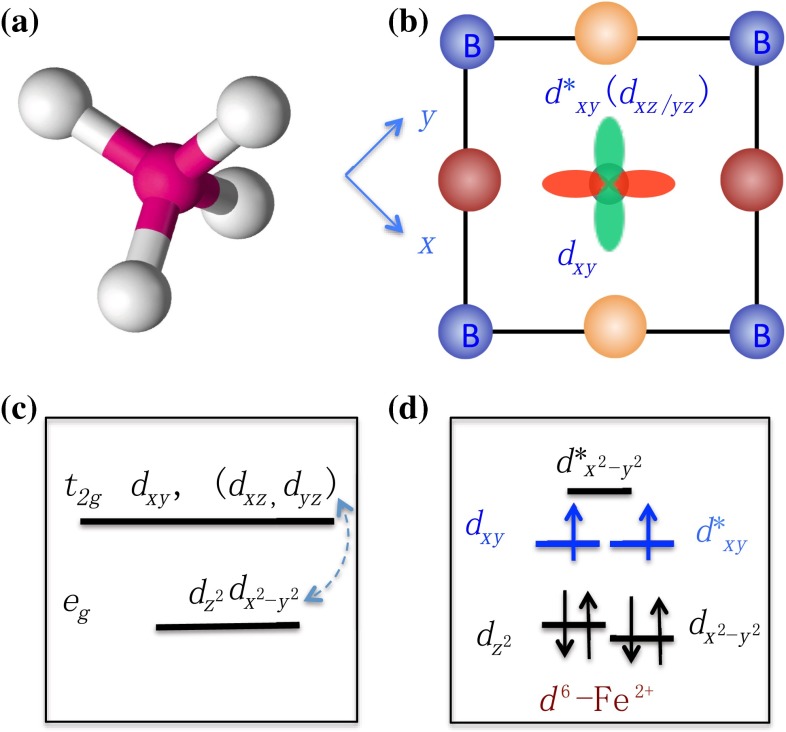
Local electronic environment and selected orbitals in iron-based superconductors. **a** The sketch of an tetrahedral complex; **b** The coupling configurations of the selected $d_{xy}$-type of orbitals to anion atoms in FeAs/Se layers; **c** The crystal field splitting of cation d-orbitals in an tetrahedral complex; **d** The local energy configurations at Fe sites in iron-based superconductors (the blue orbitals are isolated in $d^6$ filling configuration to dominate electronic physics near Fermi energy)

The electronic physics of iron-based superconductors locates on the two dimensional FeAs/Se layers. The layers are constructed by edge-shared tetrahedral $\hbox {FeAs}_4(\hbox {Se}_4)$ complexes shown in Fig. 4a. The four coordination tetrahedral complex, just slightly less popular than the octahedral complex, is another important structure unit to form crystal lattices.

In a tetrahedral complex, as shown in Fig. 4c, the $$t_{2g}$$ orbitals have higher energy than the $$e_g$$ orbitals because of their strong couplings to anions. Under such a configuration, one may jump to argue that a $$d^7$$ filling configuration can make all $$t_{2g}$$ orbitals near half-filling to satisfy the gene requirements. However, the argument is misleading because of the following two major reasons. First, the crystal field energy splitting in a tetrahedral complex between the $$t_{2g}$$ and the $$e_g$$ orbitals is much smaller than the one in the octahedral complex. Second, the $$d_{x^2-y^2}$$$$e_g$$ orbital has very large dispersion due to the short NN Fe–Fe distance in FeAs/Se layers. Therefore, the simple argument can not exclude $$d_{x^2-y^2}$$$$e_g$$ orbitals near the Fermi energy.

However, if we carefully examine the 2-Fe unit cell, because of the short distance between two NN Fe atoms, the local electronic environment of an Fe atom is not only affected by the four surrounding As/Se atoms in the tetrahedral complex but also the four neighboring Fe atoms. In fact, the $$d_{xz}$$ and $$d_{yz}$$ orbitals are strongly coupled to the $$d_{x^2-y^2}$$$$e_g$$-orbitals of the neighboring Fe atoms. Thus, a more complete picture is that the $$d_{xz}$$ and $$d_{yz}$$ orbitals form two molecular orbitals. One of them, which has $$d_{x^2-y^2}$$ symmetry character, strongly couples to the $$d_{x^2-y^2}$$$$e_g$$-orbitals of the neighboring Fe atoms. The coupling pushes this orbital to higher energy. The other, which has $$d_{xy}$$ symmetry character, remains to a pure orbital with strong couplings to the surrounding As/Se atoms. Therefore, the more accurate local energy configuration is given by Fig. 4d, in which there are two $$d_{xy}$$ type of orbitals in the middle in which one of them is formed by $$d_{xz/yz}$$ orbitals. These two orbitals can host possible high $$T_{\rm c}$$ superconductivity. With this configuration, we immediately determine that the $$3d^{6}$$ configuration of Fe$$^{2+}$$ is special to satisfy the gene requirements.

The above energy configuration has been hidden behind the simplified effective two-orbital models constructed for iron-based superconductors [22]. Near Fermi energy, the two-orbital effective model was shown to capture the band dispersions of the five-orbital models that was derived by fitting DFT calculations [23, 24]. If we check the symmetry characters of the two orbitals in the two-orbital model, both of them have $d_{xy}$ symmetry characters rather than $d_{xz/yz}$ interpreted in the original paper [22].

The above analysis suggests that the electronic structure in iron-based superconductors realizes the high $T_{\rm c}$ gene. As a matter of fact, we also notice that there are a variety of materials based on other transition metal elements with identical structures to iron-based superconductors. However, none of them exhibits high $T_{\rm c}$ superconductivity.

## The answer to the third question

A clear message from above discussion is that the genes of high $T_{\rm c}$ superconductivity stem from very special collaborations between the local electronic physics of cation–anion complexes and crystal structures. We can argue that symmetry play the key role behind the collaboration. In fact, we can argue that it is the symmetry collaboration between local complex and global crystal structures to make it possible to realize high $T_{\rm c}$ genes.

### Octahedral/tetrahedral complexes and square lattice symmetry

Both octahedral and tetrahedral complexes have a fourfold rotation principal axis. Their *d*-orbitals are classified locally by $C_4$ and $S_4$ rotation symmetries respectively. If a d orbital can be isolated in a band structure, it should have a similar classification in constructed crystal structures. This argument suggests that a square lattice symmetry is required to fulfill the gene conditions for materials constructed by octahedral and tetrahedral complexes. Both cuprates and iron-based superconductors indeed have square lattice symmetry. The selected orbitals that produce high $T_{\rm c}$ genes are classified identically in the symmetry groups of the crystal lattices and their local complexes. This correspondence allows them to be isolated in the electronic structures near Fermi energy without messing up with other orbitals.

The octahedral or the tetrahedral complexes are the most common structures in nature. They can form many different two dimensional crystal lattices. If we consider crystal structures formed by these complexes beyond the square lattice symmetry, such a correspondence is absent and different orbital characters generally get mixed. Thus, it is difficult to make the targeted orbitals to be isolated in band structures of non-square lattices formed by these two complexes, such as trigonal or hexagonal lattice structures, to fulfill the gene conditions. This explains why cuprates and iron-based superconductors are close to be unique systems to host the high $T_{\rm c}$ genes in materials constructed by octahedral and tetrahedral complexes.

### Prediction of trigonal/hexagonal high $T_{\rm c}$ electronic environments

**Fig. 5:**
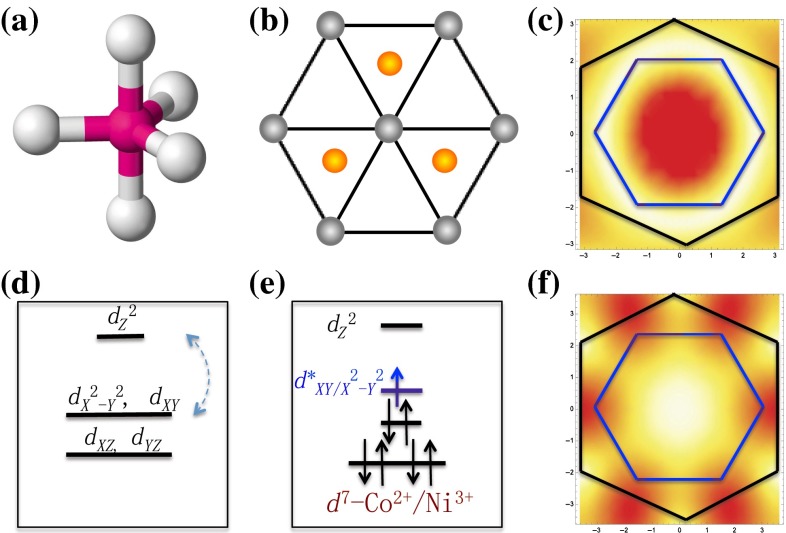
Predicted Co/Ni-based hexagonal lattices constructed by trigonal bipyramidal (TBP) complexes: **a** The sketch of a TBP complex; **b** The two-dimensional hexagonal layer formed by TBP complexes; **c** The weight distribution of an extended s-wave and Fermi surfaces (red color indicates large absolute values); **d** The crystal field splitting of cation d-orbitals in a TBP complex; **e** The local energy configurations at cation Co/Ni sites in an hexagonal layer; **f** similar to (**c**), the weight distribution of $d\pm {\mathrm{i}}d$-wave and Fermi surfaces

The symmetry collaboration argument suggests that if we want to create a high $T_{\rm c}$ gene in trigonal/hexagonal lattice structures, we may have to search lattices built by cation–anion complexes with threefold or sixfold principal rotation axis. Thus, we examine trigonal bipyramidal complexes (TBP) shown in Fig. 5a, which is a five coordination complex and carries a threefold principal rotation axis. The two dimensional hexagonal structure formed through conner-shared TBP, shown in Fig. 5b, has appeared in Mn-based $\hbox {YMnO}_3$ [25, 26] and Fe-based $\hbox {Lu}_{1-x}\hbox {Sc}_x\hbox {FeO}_3$ [27].

The explicit prediction is that a $d^7$ filling configuration, which can be realized by Co$^{2+}$ or Ni$^{3+}$ cations, fulfills the gene conditions of high $T_{\rm c}$ superconductivity in a material that carries above two dimensional hexagonal layers. Moreover, the pairing symmetry selection rule predicts that the superconducting states in these materials close to the $d^7$ filling configuration have a $d\pm {\mathrm{i}}d$ pairing symmetry.

The crystal field energy splitting of the *d*-orbitals in TBP is shown in Fig. 5. The $d_{z^2}$ orbital has the highest energy due to its strong couplings to the two apical anions. The double degenerate $d_{x^2-y^2}$ and $d_{xy}$ orbitals are strongly coupled to the in-plane anions. The double degenerate $d_{xz}$ and $d_{yz}$ orbitals have the lowest energy and are only weakly coupled to anions. As the hexagonal lattice is formed by three corner-shared TBPs, the $d_{x^2-y^2}$ and $d_{xy}$ orbitals form two molecular orbitals. One of them can strongly couple to the $d_{z^2}$ orbital so that the degeneracy is lifted. As the $d_{z^2}$ orbital has higher energy, the coupling lowers the energy level of the coupled molecular orbital. The other is completely isolated from other orbitals and can be selected to provide the desired high $T_{\rm c}$ electronic environment. A local energy configuration is described by Fig. 5e. The $d^7$ filling configuration can fulfill the gene conditions. The DFT calculation on such a structure confirms this picture [3].

Around the $d^7$ filling configuration, a quasi two dimensional band structure is formed and electronic physics near Fermi energy is dominated by a single band attributed to the selected orbital. The band has a Fermi surface shown in Fig. 5c, f. If we apply the pairing symmetry selection rule, as the pairing should be dominated on the NN bonds in the cation trigonal lattice, for the extended *s*-wave pairing, the form factor of the gap function in the momentum space is given by $\Delta _{\mathrm{s}}\propto {\mathrm{cos}}k_y+2{\mathrm{cos}}\frac{\sqrt{3}}{2}k_x{\mathrm{cos}}\frac{1}{2}k_y$, and for the $d\pm {\mathrm{i}}d$-wave pairing, the factor is given by $\Delta _{\mathrm{d}} \propto {\mathrm{cos}}k_y-{\mathrm{cos}}\frac{\sqrt{3}}{2}k_x{\mathrm{cos}}\frac{1}{2}k_y\pm {\mathrm{i}} \sqrt{3} {\mathrm{sin}}\frac{\sqrt{3}}{2}k_x {\mathrm{sin}}\frac{1}{2}k_y$. Figure 5c, f illustrates the overlap between the amplitude of the two form factors with Fermi surfaces. The degenerate $d\pm {\mathrm{i}}d$-waves collaborate well with Fermi surfaces near half filling. Therefore, the system supports a robust $d\pm {\mathrm{i}}d$-wave pairing superconducting state.

The superconducting transition temperature can be estimated by comparing the energy scales of the couplings between cations and anions in complexes. The Cu–O couplings in the octahedral complex of cuprates are more than twice stronger than the Fe–As/Se couplings in the tetrahedral complex of iron-based superconductors. The ratio of the maximum $T_{\rm c}\hbox {s}$ observed in these two families is in the similar order. In the TBP complex, the coupling strength sits between them and is about 2/3 of those in cuprates. Thus, the maximum $T_{\rm c}$ that can be realized in the TBP structure is expected to be around 100 K as the maximum $T_{\rm c}$ in cuprates can reach 160 K.

Materials constructed by the TBP complexes are very limited. The Co/Ni based materials described above have not been synthesized. Thus, it is an explicit prediction to be tested in future experiments.

## Discussion

The above prediction, if verified, justifies our answer to the first question. But most importantly, the verification can open the door to theoretically design and search for new unconventional high $T_{\rm c}$ superconductors. A general search procedure can be: (1) design a possible lattice structure that can be constructed by certain cation–anion complexes; (2) use symmetry tools to understand local electronic physics; (3) perform standard DFT calculations to obtain band structures and its orbital characters; (4) apply the gene requirements to determine conditions and likelihood on the existence of high $T_{\rm c}$ superconducting environment; (5) design realistic materials whose lattice structures can be stabilized.

In designing electronic environments for high $T_{\rm c}$ superconductivity, there are helpful clues and possible directions. For example, we can ask whether we can design crystal structures for all 3*d* transition elements to realize high $T_{\rm c}$ superconductivity. As the *d*-orbitals which are responsible for high $T_{\rm c}$ superconductivity must make strong couplings to anion atoms, they typically gain energy in the crystal field environment, which explains why all high $T_{\rm c}$ superconductors, including predicted Co/Ni-based materials, are involved with the second half part of the 3*d* transition elements in the periodic table. Whether we can overcome this limitation to make specific designs for the first half 3*d* transition elements, in particular, Mn and Cr, is a very intriguing question. Another example is to ask whether we can design superconducting states with particular pairing symmetries as we have explicit rules to determine pairing symmetries. We have noted that cuprates and iron-based superconductors are examples of the d-wave and the extended s-wave pairing symmetry in a square lattice. Our predicted material is a realization of the d-wave pairing symmetry in trigonal/hexagonal lattice structures. Thus, for example, we can ask a specific question about how to realize an extended s-wave in the trigonal/hexagonal lattice structures.

We have mainly focused on the 3*d* orbitals which are known to produce the strongest correlation effect. However, even if carrying less electron–electron correlation effect, we can also consider other type of orbitals at cation sites, including 4*d*, 5*d*, 4*f*, 5*f* and even higher level *s*-orbitals. We can search for materials as potential unconventional superconductors in which the kinematics of these orbitals near Fermi energy can be isolated and is generated through strong couplings to the *p*-orbitals of anions. In general, as long as there is a charge transfer energy gap between orbitals of cations and anions, the AFM superexchange coupling should be generated. Thus, moderate high $T_{\rm c}$ may be achieved. For 5*d* and 5*f* orbitals, because of large spin orbital couplings, the orbitals can be reconfigured to have drastically different real space configurations. This may result in more possible designs on crystal lattice structures to generate superconducting states. One example is $\hbox {Sr}_2\hbox {IrO}_4$ [28], which can be considered as a lower-energy scale clone of cuprates [29, 30]. For the *s*-orbitals, as they are symmetric in space, we may design a cubic-type three dimensional lattice structure to achieve the conditions.

In summary, cuprates and iron-based superconductors can be unified in a framework based on repulsive interaction or magnetically driven high $T_{\rm c}$ mechanisms. This unification leads to important rules to regulate electronic environments required for unconventional high $T_{\rm c}$ superconductivity. The rules can guide us to search for new high $T_{\rm c}$ superconductors. Following these rules, we made an explicit prediction about the existence of high $T_{\rm c}$ superconductivity in the Co/Ni-based two dimensional hexagonal lattice structure constructed by trigonal bipyramidal complexes. Verifying this prediction can pave a way to establish unconventional high $T_{\rm c}$ mechanism.
